# Level of interleukin-35 in patients with idiopathic membranous nephropathy and its predictive value for remission time

**DOI:** 10.3389/fimmu.2022.926368

**Published:** 2022-08-02

**Authors:** Na Zhang, Haoran Dai, Xuan Dong, Wenbin Liu, Hanxue Jiang, Qihan Zhao, Yu Gao, Zhendong Feng, Zhaocheng Dong, Yuehong Hu, Guangrui Huang, Hongliang Rui, Baoli Liu

**Affiliations:** ^1^ Beijing Hospital of Traditional Chinese Medicine, Capital Medical University, Beijing, China; ^2^ School of Traditional Chinese Medicine, Capital Medical University, Beijing, China; ^3^ Shunyi Branch, Beijing Hospital of Traditional Chinese Medicine, Beijing, China; ^4^ School of Life Sciences, Beijing University of Chinese Medicine, Beijing, China; ^5^ Pinggu Hospital, Beijing Hospital of Traditional Chinese Medicine, Beijing, China; ^6^ Beijing Institute of Chinese Medicine, Beijing, China

**Keywords:** idiopathic membranous nephropathy, Mahuang Fuzi and Shenzhuo decoction, IL-35, nephrotic syndrome, regulatory T cells

## Abstract

**Objective:**

As a member of interleukin-12 family, interleukin-35 (IL-35) plays an important regulatory role in immune response. The relationship between IL-35 and idiopathic membranous nephropathy (IMN) is still unclear, and the purpose of this study is to clarify the relationship between IL-35 and disease activity and remission of IMN.

**Methods:**

This study was a single-center, retrospective study in which all patients were diagnosed with IMN by renal biopsy or aPLA2R titer and treated with Mahuang Fuzi and Shenzhuo Decoction (MFSD). A follow-up was conducted with the endpoint of clinical complete or partial remission (CR+PR). Levels of serum IL-35 were measured and its relationship with IMN remission were analyzed. The regulatory T cell (Treg) and inducible IL-35 producing Tregs (iTR35) in peripheral blood of IMN patients were detected by flow cytometry.

**Results:**

A total of 76 IMN patients (age 51.95 ± 13.29) were followed-up for 18 (12, 24) months. The level of serum IL-35 in all patients increased after treatment, but the degree of increase in remission group was significantly higher than that in no remission (NR) group (117.6% *vs* 83.7%, *P*<0.01). The baseline IL-35 level in remission group was higher than that in NR group (174.87 *vs*.151.87 pg/ml, *P*=0.016). Cox regression analysis showed that baseline IL-35 level was a independent risk factor for IMN remission (HR 1.081, 95%CI 1.048-1.116, *P*<0.001). Patients with baseline IL-35 lower than the lower quartile (≤145.49 pg/ml) had an average remission time twice as long as those with baseline IL-35 higher than the upper quartile (> 203.05 pg/ml) (12mon *vs*. 24mon, *P*<0.01). The baseline IL-35 can predict the remission time of IMN patients with either aPLA2R positive (AUC=0.673) or negative (AUC=0.745). Analysis of 18 patients with IMN showed that IL-35 level had a higher correlation with iTR35, but not Treg (r=0.613, *P*<0.05).

**Conclusions:**

The level of IL-35 in patients with IMN showed an increasing trend with the progress of treatment, and the baseline IL-35 could predict the remission time of IMN patients, including those patients with negative aPLA2R. The level of IL-35 is related to the number of iTR35 cells.

## Introduction

Membranous nephropathy is a kind of immune-mediated glomerular disease, which is characterized by immune deposits in glomerular basement membrane and its diffuse thickening. It is one of the main pathological types of nephrotic syndrome (1). Clinically, about 20% of membranous nephropathy is secondary to infectious diseases (hepatitis B, hepatitis C, etc.), autoimmune diseases (systemic lupus erythematosus, Sjogren’s syndrome, etc.), tumors, drug side effects or harmful substances (mercury, formaldehyde), which are called secondary membranous nephropathy. About 80% of membranous nephropathy lacks a definite cause, which is called primary membranous nephropathy or idiopathic membranous nephropathy (IMN) (2). In 2009, Beck et al. (3) detected the co-location of M-type phospholipase A2 receptor (PLA2R) and IgG antibody in renal pathological sections of IMN patients, and detected the existence of PLA2R autoantibodies in circulation, thus identifying PLA2R as the main autoantigen of IMN. Thereafter, many kinds of IMN autoantigens, including THSD7A (4), NELL-1 (5), SEMA3B (6), PCDH7 (7), HTRA1 (2, 8), were identified. The discovery of these autoantigens deepened our understanding of the pathogenesis of MN.

At present, PLA2R is the main autoantigen of IMN patients, accounting for about 70% (5). There is a close relationship between the level of anti-PLA2R (aPLA2R) titer and the disease state of IMN. The KDIGO guideline takes aPLA2R titer as an important indicator to evaluate the disease state of IMN patients, which can be used to guide clinical treatment by monitoring patients’ aPLA2R titer (9). Furthermore, the change of aPLA2R titer is closely related to IMN disease activity. The faster the decline of aPLA2R titer, the shorter the remission time (10). A number of existing IMN clinical studies, including GEMRITUX, STRMEN and MENTOR studies, use the negative conversion of aPLA2R titer as a sign of IMN patients’ immunological remission (11). IMN patients’ immunological remission is earlier than clinical remission. A few months after the decrease of aPLA2R titer, patients’ proteinuria decreased significantly and gradually reached clinical remission. This phenomenon indicates that there is a causal relationship between immunological remission and clinical patients (12). However, for IMN patients with negative aPLA2R, there is still a lack of suitable indicators to evaluate their immune status and predict the clinical remission.

Interleukin-35 (IL-35), as a member of IL-12 family, is a heterodimer composed of two chains, EBI3 and p35 (13). Although the structure of IL-35 has homology with other members of IL-12 family (IL-12, IL-23, and IL-27), for example, EBI3 comes from the α chain of IL-27, and p35 comes from the β chain of IL-12, but IL-35 is significantly different from other IL-12 in function and mainly plays an immunosuppressive role (13). IL-35 is mainly produced by regulatory T cells (Treg) in humans. It is one of the main cytokines secreted by Treg cells, and it is also the key cytokine for Treg cells to exert negative immune regulation (14, 15). In addition, IL-35 can induce the generation of inducible IL-35 producing Tregs (iTR35), which is a new subset of Treg cells with immunoregulatory properties (16). The iTR35 cells can secrete more IL-35, thus forming a positive feedback loop (16). When this positive feedback loop is interrupted, the negative immunoregulation of Treg cells will decrease, and the occurrence of autoimmune reaction will be promoted.

Recent studies have shown that the level of serum IL-35 is related to autoimmune diseases. In rheumatoid arthritis (RA), serum IL-35 was negatively correlated with disease activity, and the level of IL-35 increased after remission. The level of IL-35 also increased in RA patients who received immunosuppressive therapy (17). The animal experiments show that IL-35 treatment can inhibit the clinical manifestations of collagen-induced arthritis and prevent further deterioration of the disease, suggesting that IL-35 plays an important role in the immunosuppression of RA (18). It is possible that IL-35 can improve RA by increasing the amplification of Treg cells and inhibiting the activity of Th17 cells, thus inhibiting excessive autoimmune reaction (17). In systemic lupus erythematosus (SLE), the level of serum IL-35 in active patients is lower than that in inactive patients. In addition, serum IL-35 of LN patients was significantly lower than that in SLE patients without nephritis, suggesting that IL-35 was related to renal involvement in SLE patients (19).

There are few studies on IL-35 and IMN. Roccatello et al. (20) reported that after treated with rituximab, serum IL-35 level of IMN patients increased correspondingly after remission. During this study, we analyzed the changes of IL-35 in IMN patients before and after treatment, and confirmed that the level of IL-35 increased after remission. Furthermore, we found that there was a significant difference in baseline IL-35 levels between patients with and without remission. Patients with higher baseline IL-35 levels had significantly shorter remission rates and remission times than those with lower levels of IL-35. Cox regression showed that baseline IL-35 levels were independent risk factors for IMN patients, and IL-35 levels could be used to predict remission times of IMN patients. Lastly, the level of IL-35 is related to the number of iTR35.

## Materials and methods

### Patient registration and follow-up

This study is a retrospective cohort study, including 76 IMN patients who were treated in Beijing Hospital of Traditional Chinese Medicine from Oct, 2016 to Oct, 2021. The inclusion and exclusion criteria were as follows: (1) Patients with MN diagnosis by renal biopsy and/or positive aPLA2R with nephrotic syndrome were included. (2) Exclude MN patients secondary to infectious or other systemic autoimmune diseases, or complicated with serious diseases or dysfunction of other organs (21). The remission of the study refers to the compound outcome of partial remission and complete remission. Based on the international clinical practice guideline (KDIGO), partial remission is defined as proteinuria < 3.5g/24h and a decrease of ≥50% compared with baseline, with improved or normal blood albumin and stable blood creatinine. Complete remission is defined as proteinuria < 0.3g/24h, accompanied by normal serum albumin and creatinine (9). Proteinuria > 3.5g and a decrease of < 50% compared with baseline is defined as active period. After Mahuang Fuzi and Shenzhuo Decoction (MFSD) treatment, those who do not achieve partial or complete remission are called unresponsive (22). Demography, clinical and laboratory data are collected from hospital records and reviewed by experienced doctors. The estimated glomerular filtration rate (eGFR) was calculated using the CKD-EPI equation (23).

This study was approved by the Ethics Committee of Beijing Hospital of Traditional Chinese Medicine affiliated to Capital Medical University (approval number: SQ2019YFC170377), and all the participants signed the informed consent form.

### Blood collection

A total of 13ml venous blood of each IMN patient were collected. Of these, 3ml was used for the detection of serum IL-35, and the other 10ml were placed in heparinized test tubes for isolation of mononuclear cells. Peripheral blood mononuclear cells (PBMCs) were isolated from heparinized peripheral blood by Lymphoprep™ (Axis-Shield, Alere Technologies AS, Oslo, Norway) density gradient centrifugation, following the standard protocol.

### Detection of serum IL-35 and aPLA2R titer

Serum IL-35 were assayed using a human IL-35 ELISA kit (Novus, CO, USA) according to user manual. The IL-35 ELISA kit has a dynamic range of 15.63-1000 pg/mL, and a sensitivity of 1 pg/mL. Detection of serum aPLA2R titer was performed using ELISA kit (EUROIMMUN, Lübeck, Germany) according to the manufacturer’s recommendation, a value ≥14 RU/ml was considered as positive. ELISA measurement of all samples was carried out twice and then calculate average value.

### Flow cytometry analysis

The number of Treg and iTR35 cells were performed by Flow cytometry. Treg cells were defined as CD3^+^CD4^+^CD25^+^Foxp3^+^T cells. iTR35 cells were defined as CD3^+^CD4^+^Foxp3^-^EBI3^+^IL-12p35^+^T cells. For the intracellular cytokine analysis, PBMCs were stimulated with Leukocyte Activation Cocktail (BD, USA, Cat# 550583) at 37°C for 6 hours prior to staining. Cells were surface stained with anti-CD3-PE-Cy7, anti-CD4-FITC, anti-CD25-BV421 (BD, USA, #563423; #555346; #562442) at 4°C for 30 min. And then further fixed and permeabilized (BD Biosciences, USA, #562574). Subsequently, the cells were intracellular stained with anti-Foxp3-APC, anti-EBI3-PE, anti-IL-12p35-Percp (eBioscience, USA, #17-4776-42; 12-7358-42 and MA5-23622) at 4°C for 30 min. Before detection, the living and dead cells were identified by Zombie NIR Fixable Viability Kit (Bio Legend, 423105). All samples were processed by FACSCanto™ II (BD Biosciences, USA).

### Statistical analysis

Continuous variables that conform to normal distribution are described by mean standard deviation, while those that do not conform to normal distribution are described by median and interquartile spacing. T-test of independent sample is used to compare the difference between two groups of normal distribution, and Mann-Whitney U test is used to compare the difference between two groups of non-normal distribution. The classified variables were expressed by ratio, and the comparison between the two groups was made by chi-square test. The correlation analysis adopts Pearson (bivariate are normal data) correlation analysis and Spearman (bivariate do not satisfy normal distribution) rank correlation analysis.

The ROC analysis was used to compare the area under the curve between serum IL-35 and aPLA2R titer, to evaluate its predictive ability of clinical response (the compound outcome of partial remission and complete remission) as an immune response index. Remission status analysis was performed using Kaplan–Meier method. Log-rank test was used to statistically compare the curves and *p* value. The Cox regression analysis establishes the prediction model of progress to the outcome.

For statistical analysis the SPSS statistical software package was used (SPSS 23, SPSS Inc. Chicago IL, USA). The graphs were performed by GraphPad Prism 9 (GraphPad Software Inc, USA). Some data visualization and statistical analysis were done using R, including the ggplot2 package. *P* < 0.05 was considered statistically significant.

## Results

### Levels of serum IL-35 in IMN patients

**Figure 1:**
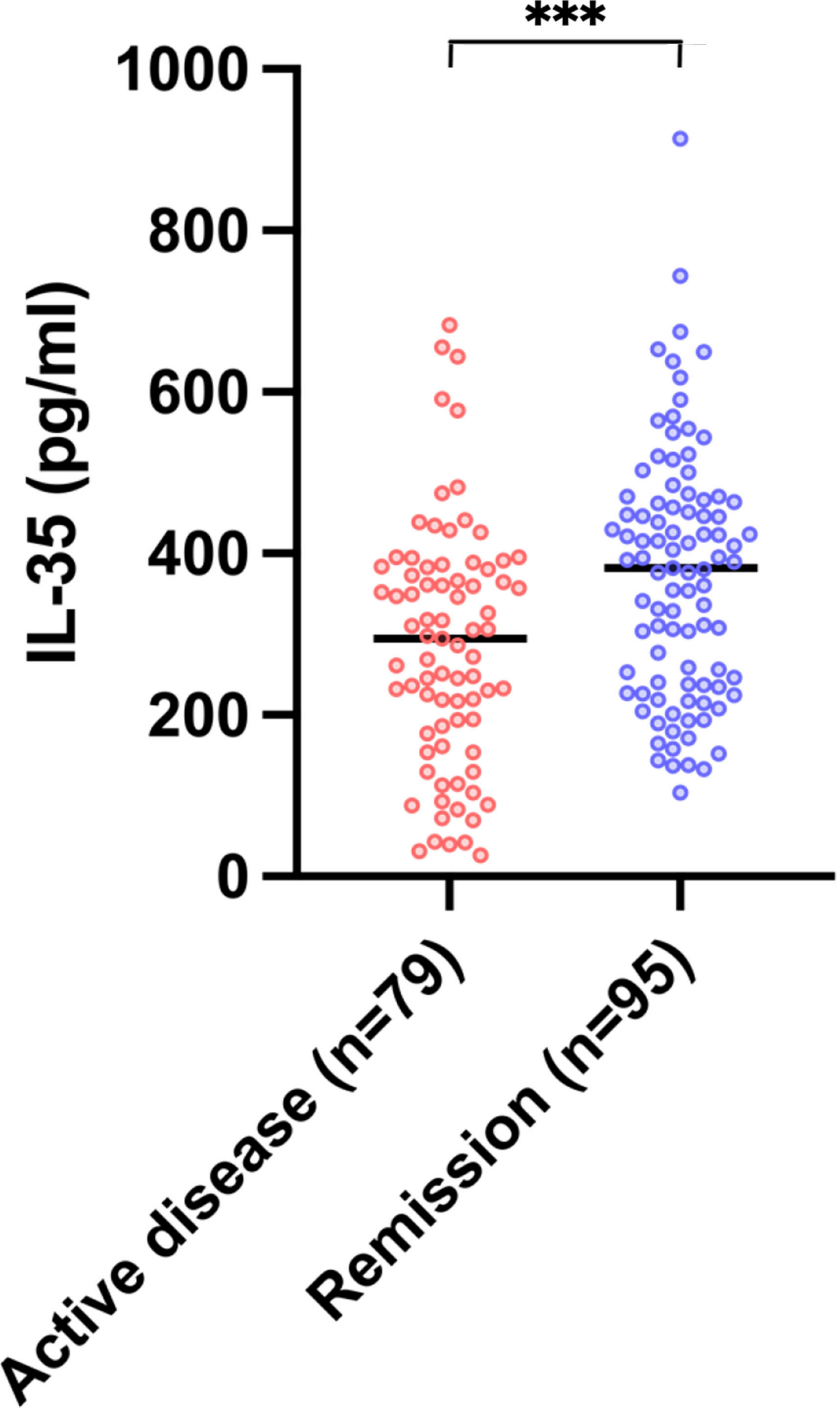
Serum IL-35 levels in IMN patients. Serum samples from 174 IMN patients were used to measure the concentration of IL-35. They were divided into two groups according to the disease remission. Active disease (n=79) defined as proteinuria >3.5g with <50% reduction from baseline; Remission (n=95) defined as the total of partial and complete remission. The data is expressed by median. *** represent *P*<0.001. IL-35, Interleukin 35.

To investigate the level of serum IL-35 in IMN patients, we conducted a cross-sectional survey. Serum samples of 174 patients with IMN were measured for serum IL-35 (**Figure 1**). According to the patient’s clinical status, these samples were divided into active disease and remission stage, and characteristics of patients at IL-35 detection were listed in **Table S1** (**Supplementary data**). It showed that the level of IL-35 in remission stage was significantly higher than that in active disease (*P*<0.05). In addition, serum IL-35 levels of 22 healthy controls were also detected, and it was found that IL-35 levels of IMN patients were significantly higher (*P*<0.05) than those of healthy controls (see **Table S2** and **Figure S1**; **Supplementary data**).

### The relationship between serum IL-35 levels and IMN disease status

**Figure 2:**
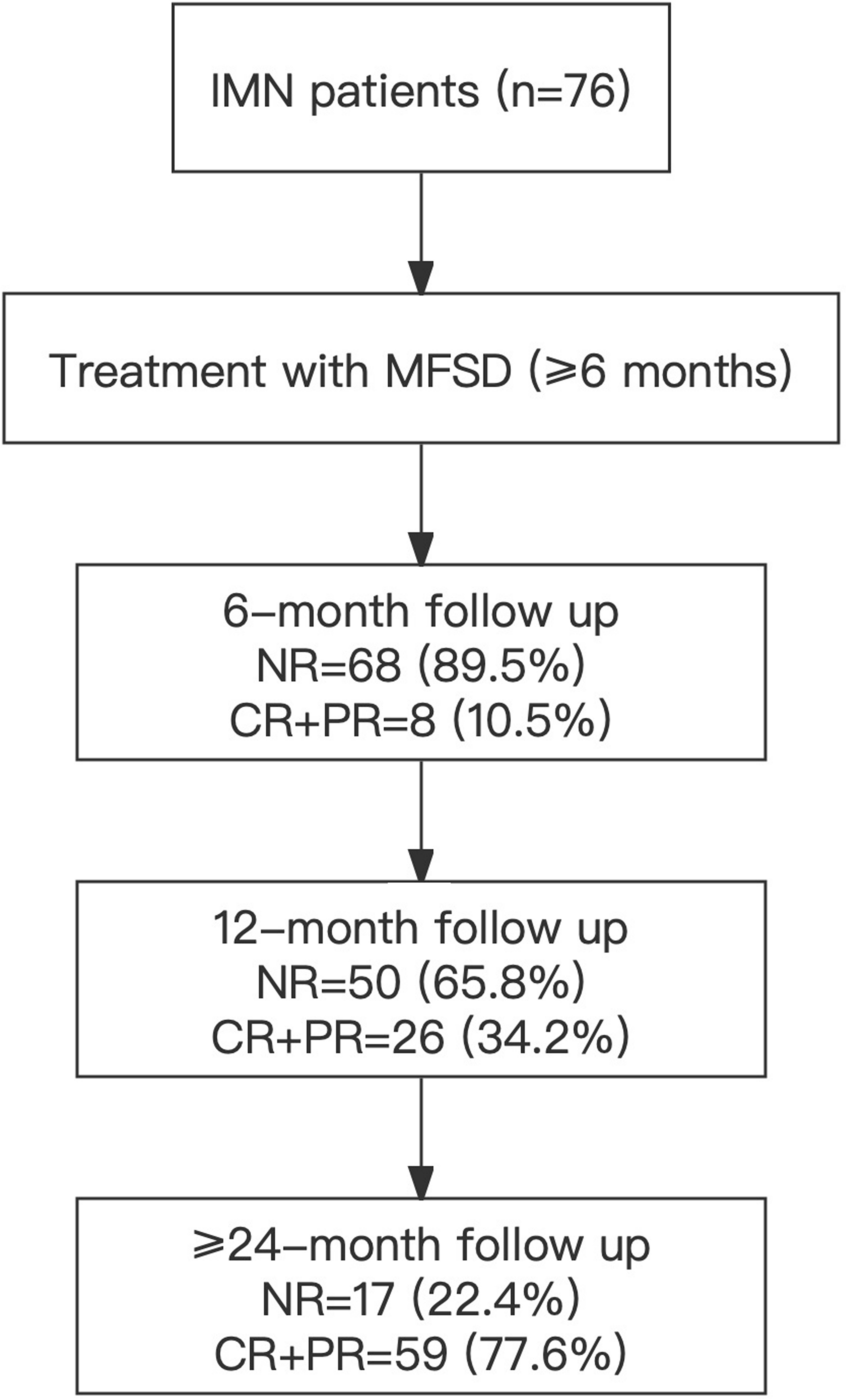
Flowchart of patients’ inclusion. IMN, Idiopathic membranous nephropathy; MFSD, Mahuang Fuzi and Shenzhuo Decoction; NR, No remission; CR, Complete remission; PR, Partial remission.

To further analyze changes in IL-35 levels during disease in patients with IMN, a cohort of 76 patients with IMN was recruited and analyzed. All patients received MFSD treatment for more than six months, with follow-up observation for 6 to 24 months or more. The patients’ disease status was evaluated at the 6th, 12th, 24th and longer months after MFSD treatment. **Figure 2** shows the trial flow chart and patient response at various time points. The results showed that at the 6th, 12th and ≥ 24th month, 10.5%, 34.2% and 77.6% of the patients got remission (CR+PR), while 89.5%, 65.8% and 22.4% of the subjects didn’t get remission (NR), respectively.

**Figure 3:**
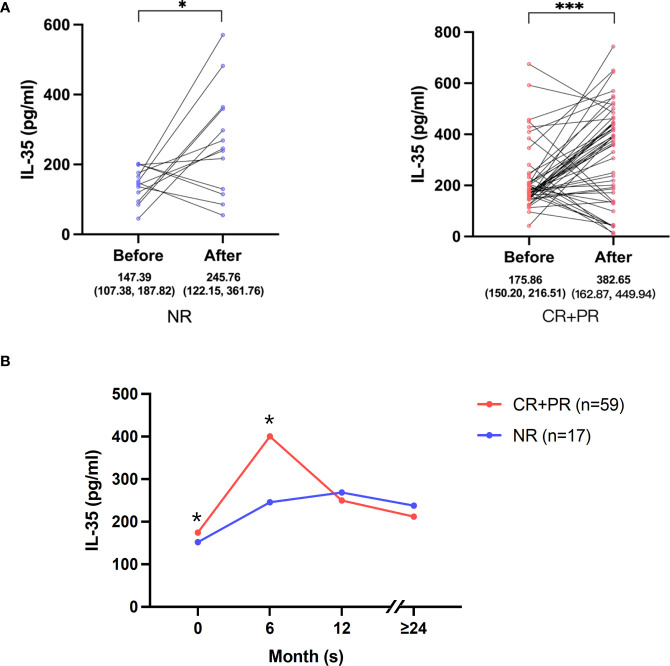
Changes of serum IL-35 levels of IMN patients **(A)** Serum IL-35 levels of each patient at baseline and follow-up endpoint were connected in parallel. IMN patients were divided into two groups according to the disease: no remission (NR) and remission (CR+PR). A Wilcoxon matched pairs signed rank test was used to compare IL-35 levels performed on the same IMN patients during active disease and in remission. *represent *P*<0.05, *** represent *P*<0.001. **(B)** Dynamic changes of serum IL-35 in IMN patients at 0, 6, 12, 24 months and above after MFSD treatment. IMN patients were divided into two groups according to the disease: no remission (NR) (n=17) and remission (CR+PR) (n=59). The data is expressed by median. *represent *P*<0.05.

According to whether they are in remission at the end of follow-up, IMN patients were divided into two groups: remission group (CR+PR) (n=59) and no remission group (NR)(n=17). Serum IL-35 levels were measured before and after treatment. Our results showed that serum IL-35 levels were 147.39 (107.38-187.82) pg/mL and 245.76 (122.15-361.76) pg/mL before and after treatment in the NR group, respectively. The CR+PR group was 175.86 (150.20-216.51) pg/ml and 382.65 (162.87-449.94) pg/ml, respectively (**Figure 3A**). It seems that serum IL-35 levels in both groups tended to increase during treatment and follow-up (*P*<0.05), while the ascensional range in IL-35 were different between the two groups. It was higher in remission group (117.6%) than that in no remission group (83.7%) (*P*<0.05). This suggested that the IL-35 level of the same IMN patient shows an increasing trend during the treatment, and the increased degree of IL-35 in the remission group is higher than that in the no remission group.

Next, we analyzed the dynamic changes of serum IL-35 in IMN patients. There was a significant difference in IL-35 level between CR+PR group and NR group at baseline and 6 months, and the IL-35 level in CR+PR group was significantly higher than that in NR group (*P*<0.05). While after 6 months, the level of IL-35 in CR+PR group gradually decreased, and the median value was like that in NR group. In NR group, the change of IL-35 level was smaller than that in CR+PR group. IL-35 level increased slightly at 0-6 months, and then gradually decreased. The levels of IL-35 in the two groups had no difference after the 12th and 24th or longer months (**Figure 3B**). In summary, the level of IL-35 in IMN patients with remission rises faster and the peak value is higher at 0-6 months than that with no remission.

### Serum IL-35 levels in IMN patients were different at baseline

Based on the dynamic changes of serum IL-35 in patients with IMN, we found the difference of baseline IL-35 levels between patients with remission and no remission, so we next analyzed the baseline data of patients. We found that compared with the no remission group, baseline serum IL-35 levels in remission group were significantly higher (*P*<0.05) than that in NR group (**Table 1**). However, the level of aPLA2R titer between the two groups was not significantly different. Besides the serum IL-35 levels, we also found that baseline 24hUTP was lower (*P*<0.01) and ALB was higher (*P*<0.01) in remission group. But there was no significant difference in other indexes (eGFR, using of immunosuppressant, etc.) (**Table 1**).

**Table 1:** Baseline characteristics of IMN patients according to disease remission.

	Total (n=76)	CR+PR (n=59)	NR (n=17)	*P* Value
Age (years)	51.95 ± 13.29	52.91 ± 14.06	42.08 ± 11.47	0.050
Gender/Male (%)	45 (59.2)	33 (55.9)	12 (70.6)	0.279
Diabetes (%)	14 (18.4)	11 (18.6)	3 (17.6)	0.926
Hypertension (%)	31 (40.8)	21 (35.6)	10 (58.8)	0.086
Nephrotic syndrome (%)	50 (65.8)	35 (59.3)	15 (88.2)	0.027
24hUTP (g/24h)	5.75 (3.78, 9.43)	5.49 (3.89, 8.20)	10.54 (4.59, 17.00)	0.001
ALB (g/L)	27.61 ± 6.79	28.02 ± 6.41	24.44 ± 6.93	0.003
TG (mmol/L)	1.96 (1.73, 2.91)	2.05 (1.75, 2.86)	2.81 (1.87, 3.59)	0.067
CHO (mmol/L)	6.82 (5.70, 8.98)	7.20 (5.60, 9.04)	7.11 (6.07, 10.41)	0.085
SCr (umol/L)	70.00 (54.60, 93.55)	64.30 (54.70, 101.00)	82.10 (66.65, 97.50)	0.114
eGFR (ml/min/1.73m^2^)	142.90 (99.15, 155.25)	145.70 (95.03, 155.85)	135.20 (99.45, 144.95)	0.222
Immunosuppressant (%)	47 (61.8)	39 (66.1)	8 (47.1)	0.154
Remission time (month)	18 (12, 24)	18 (12, 24)	12 (7.5, 18)	0.099
aPLA2R Positive (%)	47 (61.8)	34 (57.6)	13 (76.5)	0.159
aPLA2R titer (RU/ml)	100.00 (19.50, 243.42)	78.59 (21.32, 217.33)	184.90 (18.00, 269.41)	0.821
IL-35 (pg/ml)	173.54 (145.49, 203.05)	174.87 (149.83, 205.42)	151.87 (107.38, 190.82)	0.016

NR, No remission; CR, Complete remission; PR, Partial remission. Age, 24hUTP, ALB, TG, CHO, SCr, eGFR, IL-35, aPLA2R titer, Remission time these indicators are continuous variables. Other indicators are dichotomies.

### Univariate and multivariate Cox regression analysis of serum IL-35 levels in IMN patients

A Cox regression analysis of prognostic factors was utilized for single and multiple factor tests, and the dependent variable is whether IMN patients has achieved clinical remission. Depending on the demographic and clinical factors, Age, Gender, Diabetes, Hypertension, Nephrotic syndrome, 24hUTP, ALB, TG, CHO, SCr, eGFR, immunosuppressant, aPLA2R titer, and IL-35 were included univariate variables. The results showed that nephrotic syndrome, ALB, and IL-35 were associated with remission of IMN (**Table 2**). After considering the clinical factors and univariate Cox regression, we included Age, Gender, 24hUTP, ALB, eGFR and IL-35 in multivariate Cox regression model. We found that IL-35 was significantly related to the remission of IMN patients (HR 1.081, 95% CI: 1.048-1.116, *P*< 0.001), which means that for every 10 pg/ml increase in baseline serum IL-35 level, the remission rate of IMN patients increases by 8.1% (**Table 2**).

**Table 2:** Cox regression analysis for remission time in IMN patients (IL-35 for continuous variables).

	Univariate analysis		Multivariable Analysis	
	HR (95% CI)	*P* Value	HR (95% CI)	*P* Value
Age	0.997 (0.978, 1.016)	0.777	1.010 (0.988, 1.033)	0.370
Gender	1.075 (0.636, 1.815)	0.787	1.291 (0.673, 2.476)	0.443
Diabetes	1.199 (0.621, 2.315)	0.589		
Hypertension	0.826 (0.484, 1.411)	0.484		
Nephrotic syndrome	2.244 (1.307, 3.853)	0.003		
Urine protein	0.943 (0.873, 1.018)	0.130	1.057 (0.956, 1.168)	0.280
ALB	1.069 (1.028, 1.113)	0.001	1.069 (1.008, 1.134)	0.026
TG	0.999 (0.847, 1.180)	0.995		
CHO	0.946 (0.862, 1.040)	0.251		
SCr	0.993 (0.982, 1.003)	0.146		
eGFR	1.005 (0.997, 1.014)	0.184	1.006 (0.997, 1.015)	0.181
Immunosuppressant	0.702 (0.408, 1.208)	0.201		
aPLA2R titer	1.000 (0.999, 1.001)	0.917		
IL-35 per 10 pg/ml	1.092 (1.060, 1.126)	<0.001	1.081 (1.048, 1.116)	<0.001

Next, we performed another regression analysis of Cox for categorical variables based on the quartile of baseline serum IL-35 levels. Patients with IMN were divided into four groups according to median and interquartile spacing of baseline serum IL-35 levels (25%, 50%, 75%): Group1 (≤145.49pg/ml), Group2 (145.49-173.54pg/ml), Group3 (173.54-203.05pg/ml), and Group4 (>203.05pg/ml). There was no significant difference between Group2, Group3 and Group1 (*P*>0.05), while Group4 was significantly different from Group1 (HR 4.542, 95% CI 1.991-10.361, *P <*0.05) (**Table 3**). This means that the remission rate in IMN patients with baseline serum IL-35 >203.05pg/ml (Group4) was 4.542-fold higher than in the group IL-35 ≤ 145.49 (Group1). In summary, Cox regression analysis showed that IL-35 is a predictive factor for remission of IMN. In addition, ALB at baseline is also significant for predicting prognosis.

**Table 3:** Cox regression analysis for remission time in IMN patients (IL-35 for categorical variables).

	**Univariate analysis**		**Multivariable Analysis**	
	**HR (95% CI)**	***P* Value**	**HR (95% CI)**	***P* Value**
IL-35 (pg/ml) is grouped according to median with interquartile range				
Group 1 (≤145.49)	Ref		Ref	
Group 2 (145.49-173.54)	1.931 (0.881, 4.235)	0.100	1.902 (0.825, 4.384)	0.131
Group 3 (173.54-203.05)	1.556 (0.699, 3.465)	0.279	1.500 (0.662, 3.400)	0.331
Group 4 (>203.05)	4.986 (2.306, 10.783)	<0.001	4.542 (1.991, 10.361)	<0.001

IL-35 is grouped according to median with interquartile range, Group1 ≤ 145.49; 145.49 <Group2 ≤ 173.54; 173.54 <Group3 ≤ 203.05; Group4 >203.05.

### The baseline serum IL-35 levels can predict the remission time and remission rate of IMN patients

**Figure 4:**
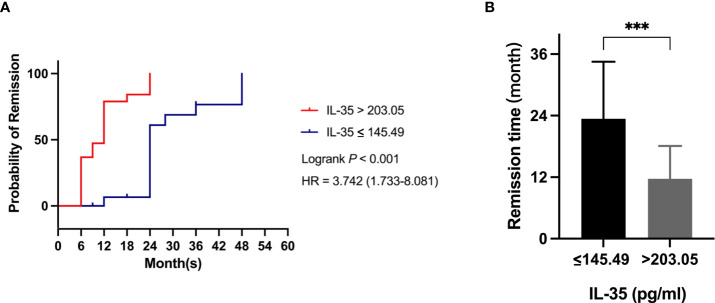
Comparison of remission time of IMN patients with different baseline IL-35 levels. **(A)** Kaplan-Meier analysis was used to assess the remission time between IMN patients of serum IL-35 ≤ 145.49pg/ml (blue line) (n=18) with that of >203.05pg/ml (red line) (n=19). **(B)** Comparison of median remission time between IMN patients of serum IL-35 ≤ 145.49pg/ml with that of >203.05pg/ml. *** represent *P*<0.001.

According to COX regression analysis, we found that there were significant differences in remission time and remission rate with IMN patients’ baseline serum IL-35 ≤ 145.49 pg/ml (Group 1) and > 203.05 pg/ml (Group 4). Kaplan-Meier survival analysis was used to compare the remission time between the two groups. The results showed a median remission time of 12 months in IMN patients with Group 1(n=18) and 24 months with Group 4 (n=19), and the remission time of group 4 is twice if that of group1 (**Figures 4A, B**). **Figure 4A** In addition, the overall remission rate in Group 4 was 3.742 times than in Group 1 (*P<0.001*, **Figure 4B**).

The remission rates of the two groups were also compared at different follow-up time points. The remission rates of Group 1 were 0, 5.6% and 61.1% at the 6th, 12th and 24th month respectively. While the Group 4 were 42.1%, 78.9% and 100% at the 6th, 12th and 24th month respectively (**Table 4**). This further indicated that baseline serum IL-35 levels are an important predictor of remission rates in patients with IMN.

**Table 4:** Remission rates at different time points in IMN patients with baseline IL-35 (pg/ml) >203.05 and ≤145.49.

	>203.05 (n=19)	≤145.49 (n=18)
6-month follow up	42.1%	0
12-month follow up	78.9%	5.6%
24-month follow up	100%	61.1%

Our results have indicated that the baseline serum IL-35 level has important predictive value for the remission time and remission rate of IMN patients. For IMN patients with lower baseline serum IL-35 levels, the remission rate was lower and the remission time was longer.

### The predictive value of IL-35 on immunological and clinical remission in patients with aPLA2R positive and negative IMN

**Figure 5:**
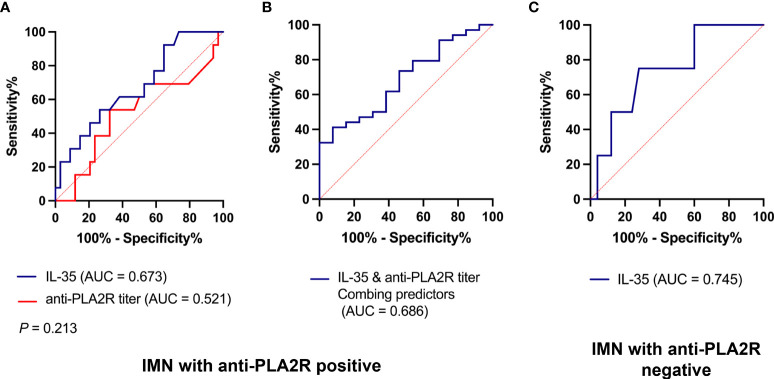
Area under the ROC curve for prediction of IMN remission. ROC compares the predictive value of IL-35 and/or aPLA2R titer in patients with IMN. **(A)** the predictive value of IL-35 or aPLA2R in IMN patients with positive aPLA2R; **(B)** the predictive value of IL-35 combined with aPLA2R titer in IMN patients with positive aPLA2R; **(C)** the predictive value of IL-35 in IMN patients with negative aPLA2R.

We next investigated the predictive effect of IL-35 on clinical response in patients with aPLA2R-positive and negative IMN. At present, there is controversy about whether the baseline aPLA2R titer can predict the clinical response in IMN patients. For IMN patients with aPLA2R positive, we used IL-35, aPLA2R titer and the combination of the two to predict the clinical response. The results showed AUC (Area Under Curve) values of IL-35 was 0.673, the aPLA2R titer was 0.521, and combined diagnosis was 0.686, suggesting that a combination of IL-35 and aPLA2R titer was more effective than IL-35 and aPLA2R titer alone (**Figures 5A, B**).

For IMN patients with negative aPLA2R, there was a previous lack of indicators to evaluate immunological remission in these patients. We used IL-35 levels to predict immunological and clinical remission in patients with negative aPLA2R, and found that IL-35 could predict remission time with an AUC of 0.745 (**Figure 5C**). For aPLA2R negative IMN patients, IL-35 had a sensitivity of 75% and a specificity of 72% in predicting remission time with a Cut-Off value of 150.9 pg/ml.

Our results suggest that IL-35 is predictive of immunological and clinical remission in both aPLA2R positive and negative IMN patients, particularly in aPLA2R negative IMN patients.

### Correlations between serum IL-35 levels and Treg and iTR35 cells frequency in PBMCs

**Figure 6:**
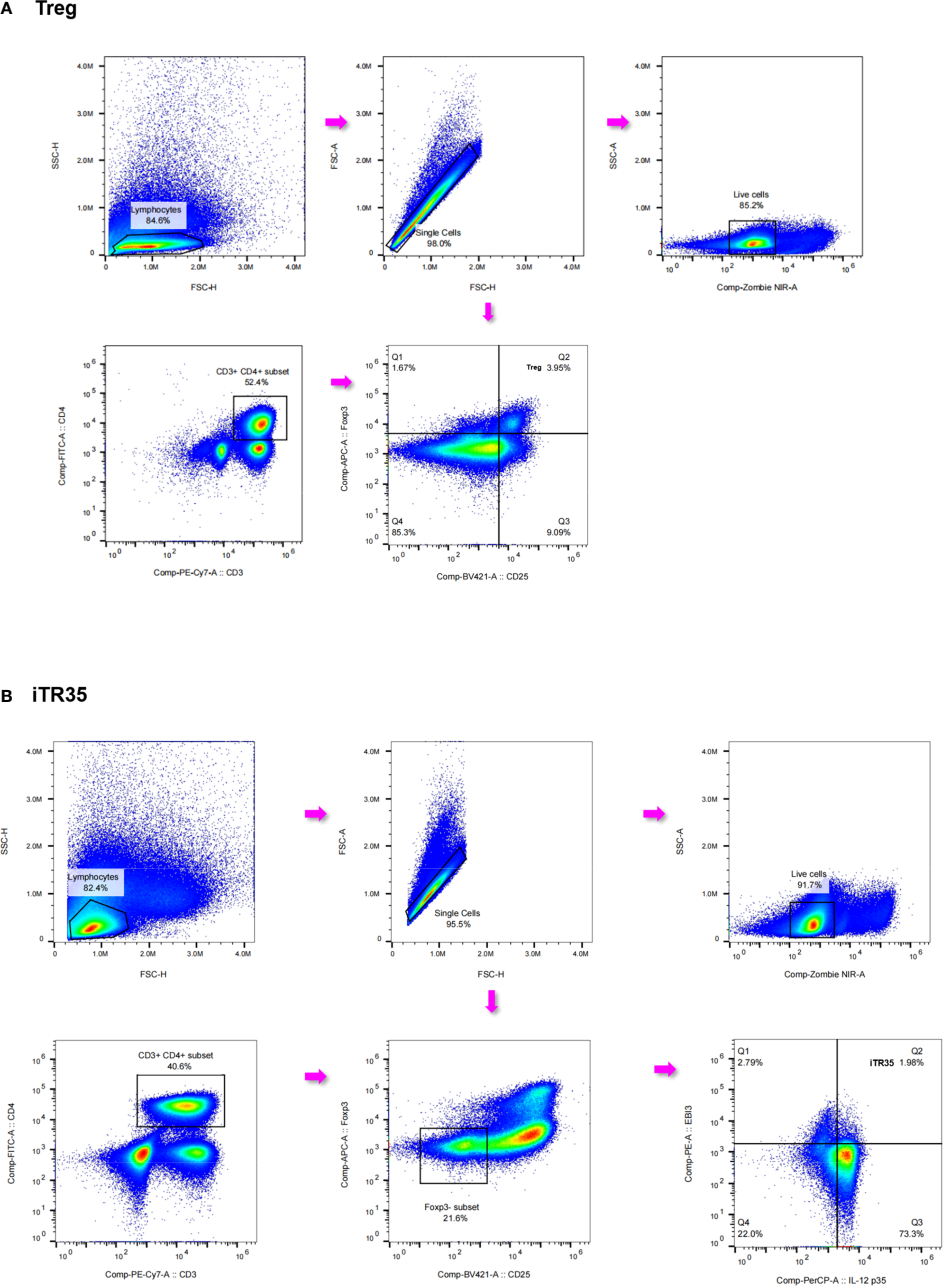
Flow gating strategy and representative diagram of iTR35 and Treg. Flow cytometric analysis of Treg and iTR35 cells frequency in the peripheral blood of IMN patients. **(A)** Treg (CD3^+^CD4^+^CD25^+^Foxp3^+^T cells) gating strategy and representative diagram of IMN patients. The picture sequence is: lymphocytes are identified in PBMC., adherent cells are removed, dead cells are removed, CD3+CD4+ cells and CD25+Foxp3+ cells. **(B)** iTR35 (CD3^+^CD4^+^CD25^-^Foxp3^-^EBI3^+^IL-12p35^+^T cells) gating strategy and representative diagram of IMN patients. The picture sequence is: lymphocytes are identified in PBMC., adherent cells are removed, dead cells are removed, CD3+CD4+ cells, CD25-Foxp3-cells and EBI3+IL-12p35+cells. SSC, side scatter; FSC, forward scatter; NIR, near-infrared; FITC, fluorescein isothiocyanate; APC, allophycocyanin; PE, phycoerythrin; EBI3, Epstein-Barr virus-induced gene 3; IL, interleukin; Foxp3,forkhead box p3; Treg, regulatory T; iTR35, IL-35-induced regulatory T; PBMC, peripheral blood mononuclear cell.

**Figure 7:**
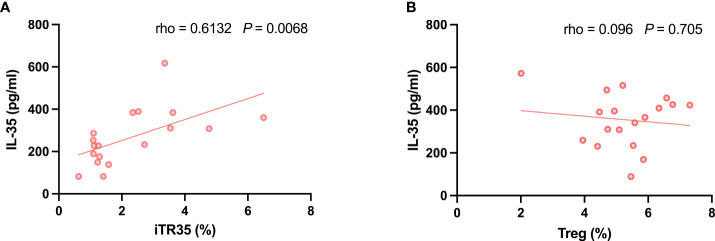
Correlation analysis of Treg、iTR35 cells and IL-35. **(A)** The positive correlation between iTR35 cells frequency and IL-35 was expressed by Spearman rank correlation analysis. **(B)** There is no significant correlation between Treg cells frequency and IL-35.

As previously described, there was an association between serum IL-35 levels and Treg and iTR35 cells frequency. We measured the frequency of Treg cells (CD3^+^CD4^+^CD25^+^Foxp3^+^T cells) and iTR35 (CD3^+^CD4^+^Foxp3^-^EBI3^+^IL-12p35^+^T cells) in PBMCs from 18 IMN patients, and the detected flow analysis image was shown in **Figure 6**; **Figure S2** and **Figure S3** (**Supplementary Data**). The correlation analysis showed that the correlation between IL-35 and Treg was not obvious (r=0.096, *P*>0.05). However, there was a positive correlation between IL-35 and iTR35 cells (r=0.6132, *P*<0.05) (**Figure 7**). This result suggested that IL-35 may exert immunosuppressive effect by regulating iTR35 cells.

Moreover, we also analyzed the correlation between IL-35 and other parameters. And we found that serum IL-35 levels were positively correlated with ALB (r=0.37, *P*<0.01) and remission (r=0.28, *P*=0.01), and negatively correlated with 24hUTP (r=-0.26, *P*=0.02), nephrotic syndrome state (r=-0.39, *P*<0.01) and remission time (r=-0.46, *P*<0.01). the detail is shown in **Table S3** (**Supplementary Data**).

## Discussion

It is considered that antibodies generated by autoimmune reaction combine with target antigens on podocytes in glomerulus to form *in situ* immune complexes, as the major pathogenic mechanism of IMN (24, 25). The autoantibodies in this process are mainly IgG4, in which humoral immunity and B cells play a key role. As an important factor of autoimmune reaction, T cells participate in the autoimmune reaction of IMN by regulating B cells differentiation and antibody production (26). The regulatory T cells are the main subsets of helper T cells that play an immunosuppressive role, which can inhibit the autoimmune reaction and maintain immune tolerance (14). Treg cells directly or indirectly inhibit immune response and antibody production by expressing cellular receptors such as CTLA-4 or secreting cytokines such as TGF-β, IL-10, and IL-35, and are the therapeutic target of autoimmune diseases (14).

According to KDIGO 2021 glomerular diseases guideline (9), the treatment of IMN mainly includes rituximab (CD20 McAb), glucocorticoid, cyclophosphamide, calcineurin inhibitor (CNI), and other immunosuppressive therapies (9). Although immunosuppressive agents can make most patients with IMN achieve clinical remission, the high recurrence rate, drug side effects, and complications caused by long-term Immunosuppressive therapy are still problems that cannot be ignored (27, 28).

Our previous clinical study confirmed that after an 18-month (12.5-30 month) follow-up, the remission rate (CR+PR) of IMN patients treated with the MFSD regimen reached 61.4% (22). Among them, the remission rate was 59.6% in patients who had received immunosuppressive therapy but no remission or terminated due to side effects and 65.5% in IMN patients who had never received immunosuppressive therapy, and there was no statistical difference between them (*P*=0.254) (22). During the follow-up of MFSD treatment, the course of treatment was significantly related to the remission of IMN (*P*<0.05). There was no obvious change in renal function in most patients before and after treatment, and no serious adverse reactions occurred in all patients during treatment (22). Thus, the MFSD regimen is effective for IMN patients with high safety, and it is also effective for patients who have not received immunosuppressive therapy, and those who have received immunosuppressive therapy without remission (22). A randomized controlled clinical study comparing the efficacy of MFSD and CNI in the treatment of IMN is underway.

Our research shows that the level of IL-35 in patients with IMN in remission stage is significantly higher than that in active disease, which may be related to the shift from immune state to immunosuppression. It is worth noting that the level of IL-35 in IMN patients is always higher than that in healthy controls, whether in remission or active period, suggesting that as an autoimmune disease, IMN patients’ autoimmune response remains active, even if the disease is relieved. Mohd shukri et al. (29) also found a similar phenomenon in SLE patients, the level of IL-35 in SLE patients was higher than that in healthy controls, but they did not further observe whether there were differences in IL-35 levels in SLE patients with different disease states.

After 6 months of MFSD treatment, the level of IL-35 was significantly higher than that of baseline, which indicated that IMN patients who could be relieved recovered quickly in the early stage of immunosuppression, so it was easy to be relieved. If the increase of IL-35 level represents the change of immune state *in vivo*, that is, from hyperimmunity to immunosuppression, then it can be expected that the remission of IMN patients will be achieved accordingly. The immune status of patients who have not been relieved has been relieved to some extent, but it still takes some time to achieve clinical remission. Roccatello et al. (20) treated IMN patients with rituximab for 12 months, about 68% of patients achieved clinical remission (CR+PR). They discovered that the level of serum IL-35 in remission patients increased significantly, which is like our results (20). They also found that the number of Treg increased significantly after 6 months of treatment, but only lasted until the 12th month in remission patients (20). Unlike their findings, they did not observe a significant increase in the level of IL-35 in patients without remission, while we did observe a small increase in the level of IL-35, although the increase was lower than that in patients with remission. This may be related to the different treatment regimens and the length of follow-up. Furthermore, we believe that the baseline IL-35 level of patients before receiving treatment is an influential factor that can’t be ignored. The baseline IL-35 level of IMN patients who achieved remission after treatment was significantly higher than that of those who did not.

The currently reported indicators used to evaluate the immune status and predict the remission time of IMN patients include the changes of aPLA2R titer (30) and BAFF levels (31). However, they are mainly used in IMN patients with positive aPLA2R, but for nearly 30% of IMN patients with negative aPLA2R, there is a lack of corresponding indicators. According to our study, the combination of IL-35 and aPLA2R titer not only improves the sensitivity of predicting the remission time of patients with aPLA2R positive IMN, but also can be used to predict the remission time of patients with aPLA2R negative IMN, which provides a good indicator for judging the immune status of patients with aPLA2R negative IMN.

The study of Rosenzwajg has shown that the amount of Treg in peripheral blood can be used to evaluate the immune status and predict the remission time of IMN patients (32). They also found that Treg cells may have changed before the decrease of aPLA2R titer, while Motavalli et al. (26) confirmed that the imbalance between Treg/Th17 plays an important role in the pathogenesis of IMN. However, there are technical difficulties in detecting the number of Treg, which limits its clinical application. As a cytokine mainly secreted by Treg, IL-35 is more economical and convenient than Treg, so it is easier to be used clinically.

What kind of T cells have a greater influence on the level of IL-35, Treg, or iTR35, a subgroup of Treg? Our research shows that the latter has a higher correlation with the level of IL-35. As a subgroup of Treg, we speculate that iTR35 directly regulates and influences the level of IL-35 (33). Studies by Wang et al. (34) in allergic asthma and Layhadi et al. (35) in allergic rhinitis also confirmed the positive correlation between iTR35 and IL-35.

In our previous studies, we also observed the therapeutic effect of MFSD in passive Hyman nephritis (PHN) rat model, but its mechanism is still under study (36). Combined with the changes of IL-35 in this study and its predictive effect on remission time, we suspect that MFSD may enhance the immune suppression ability of the body by up-regulating IL-35, which makes the immune state of IMN patients change from hyperfunction to equilibrium. The immunomodulatory ability of IMN patients was restored after MFSD treatment, which may be through remodeling the immune balance to achieve the purpose of curing the disease. This also indicates that besides B cells, regulatory T cells play an important role in the pathogenesis and treatment of IMN. Although Rituximab therapy promotes the depletion of B cells, it still takes a long time to induce IMN remission. This process is accompanied by the increase of Treg cells, suggesting that Treg cells are also involved in the role of Rituximab (20).

In conclusion, our research has confirmed the change of IL-35 in IMN patients. The baseline IL-35 level reflects the immune status of patients, and patients with higher baseline IL-35 can be relieved more quickly. We also show that the therapeutic target of the MFSD regimen may be different from the traditional immunosuppressive regimen, which provides more choices for the treatment of IMN.

## Limitation

The following shortcomings exist in this study: this study is a retrospective study, and a larger number of patients would have been more beneficial in drawing the conclusions of this study. In addition, the number of samples for detecting Treg and iTR35 in PBMC of patients with IMN is a little less, due to the difficulty in PBMC preservation.

## Data availability statement

The original contributions presented in the study are included in the article/**Supplementary material**. Further inquiries can be directed to the corresponding author/s.

## Ethics statement

This study was reviewed and approved by Medical Ethics Committee of Beijing Hospital of Traditional Chinese Medicine. The patients/participants provided their written informed consent to participate in this study.

## Author contributions

BL and HR were responsible for conception of the study. HD, YG, XD, HJ, and YH are responsible for data analysis and interpretation. WL, ZF, ZD, QZ and GH were responsible for conception of the study and responsible for critical revision of important content, and undertook part of the data analysis and interpretation work. NZ was involved in drafting the manuscript. BL and HR were responsible for approving the final version to be published and agree to be responsible for all aspects of the work and to ensure that issues relating to the accuracy or completeness of any part of the work are properly investigated and resolved. The authenticity of the original data in this paper has been confirmed by BL and HD, and they are responsible for this. All authors contributed to the article and approved the submitted version.

## Funding

This work was supported by grants from the National Key Research and Development Project of China (no. 2019YFC1709402 to BL), National Natural Science Foundation of China (no. 81973793 to BL, 82004269 to HD), and Capital’s Funds for Health Improvement and Research (No. 2020-2-2234 to BL).

## Acknowledgments

The authors sincerely acknowledge the support of Dr. Huihui Liu, Xin Liu for their support of flow cytometry for evaluation of immunity during this work.

## Conflict of interest

The authors declare that the research was conducted in the absence of any commercial or financial relationships that could be construed as a potential conflict of interest.

## Publisher’s note

All claims expressed in this article are solely those of the authors and do not necessarily represent those of their affiliated organizations, or those of the publisher, the editors and the reviewers. Any product that may be evaluated in this article, or claim that may be made by its manufacturer, is not guaranteed or endorsed by the publisher.
