# Supplementary material for: Level of interleukin-35 in patients with idiopathic membranous nephropathy and its predictive value for remission time
Source: Front Immunol. 2022 Aug 2;13:926368. doi: 10.3389/fimmu.2022.926368 (PMC9379805; doi:10.3389/fimmu.2022.926368)
Supplement: Supplementary file 1 [file DataSheet_1.pdf]

Level of interleukin-35 in patients with idiopathic membranous nephropathy and its predictive value for remission time

Na Zhang[1, 2], Haoran Dai[3], Xuan Dong[1, 2], Wenbin Liu[5], Hanxue Jiang[1, 2], Qihan Zhao[1, 2], Yu Gao[1, 2], Zhendong Feng[1, 6], Zhaocheng Dong[1, 5], Yuehong Hu[1, 2], Guangrui Huang[5], Hongliang Rui[1, 4], and Baoli Liu[1, 2]

[1]Beijing Hospital of Traditional Chinese Medicine, Capital Medical University, Beijing, China. [2]School of Traditional Chinese Medicine, Capital Medical University, Beijing, China. [3]Shunyi Branch, Beijing Hospital of Traditional Chinese Medicine, Capital Medical University, Beijing, China. [4]Beijing Institute of Chinese Medicine, Beijing, China. [5]School of Life Sciences, Beijing University of Chinese Medicine, Beijing, China. [6]Pinggu Hospital, Beijing Hospital of Traditional Chinese Medicine, Beijing, China.

Supplementary Data

Cross-sectional study of IL-35 level in IMN patients

A total of 174 blood samples from 126 IMN patients were tested for serum IL-35. The clinical characteristics of these patients when testing IL-35 are shown in **Table S1**. For the IMN patients, active disease was defined as: 24hUTP>3.5g/24h with <50% reduction from baseline; remission was defined as: 24hUTP<3.5g/24h and decreased by≥50% from baseline, improved or normal albumin, and stable creatinine.

It showed that WBC, PLT, 24hUTP, TG, CHO, HDL, LDL, UREA in active IMN patients were significantly higher than those in remission IMN patients, and the female ratio, ALB, and serum IL-35 levels were significantly lower than those in remission IMN patients. Age, IL-35, 24hUTP, ALB, TG, CHO, eGFR, ALT, AST, TP, LDL, HDL, WBC, NEUT, LYM, MONO, EO, BASO, RBC, Hgb, PLT these indicators are continuous variables. Other indicators are dichotomies.

Table S1. Characteristics of IMN patients when testing IL-35

	Total (n=174)	Active disease (n=79)	Remission (n=95)	*P* Value
Age (years)	52±13	49±14	53±13	0.057
Gender (female)	68 (39.1%)	24 (30.4%)	44 (46.3%)	0.032
WBC (10^9/L)	6.98 (5.86, 8.86)	7.45 (6.09, 10.27)	6.82 (5.78, 8.08)	0.041
Hgb (g/L)	133±17	132±18	134±16	0.239
PLT (10^9/L)	277 (239, 333)	321 (244, 382)	265 (231, 299)	0.001
24hUTP (g/24h)	3.31 (1.04, 6.61)	7.5 (4.93, 10.84)	1.22 (0.56, 2.40)	<0.001
ALB (g/L)	36.1 (28.9, 41.2)	28.3 (24.0, 33.3)	40.3 (37.4, 43.3)	<0.001
TG (mmol/L)	1.84 (1.41, 2.38)	2.01 (1.68, 3.07)	1.63 (1.31, 2.04)	<0.001
CHO (mmol/L)	5.87 (4.94, 7.31)	7.31 (5.91, 8.99)	5.19 (4.28, 5.92)	<0.001
HDL (mmol/L)	1.36 (1.14, 1.56)	1.41 (1.21, 1.68)	1.3 (1.1, 1.52)	0.007
LDL (mmol/L)	3.72 (2.82, 5.00)	4.97 (3.71, 6.22)	3.22 (2.59, 3.79)	<0.001
eGFR (ml/min/1.73m^2)	152.2 (114.2, 162.0)	149 (119.3, 158.2)	153.5 (106.3, 163.0)	0.323
UREA (mmol/L)	6.04 (4.67, 8.30)	6.56 (5.04, 8.78)	5.65 (4.42, 7.53)	0.049
UA (μmol/L)	385.4 (323.3, 434.1)	384.5 (335.7, 428.8)	388.5 (316.6, 444.3)	0.855
IL-35 (pg/ml)	338.63 (218.95, 428.85)	294.23 (177.05, 382.53)	382.22 (237.43, 463.94)	<0.001

Abbreviation 24hUTP: 24-hour urinary protein quantity; ALB: Albumin; TG: Triglyceride; CHO: Cholesterol; UA: Uric Acid; eGFR: estimated Glomerular Filtration Rate; ALT: Alanine Aminotransferase; AST: Aspartate Aminotransferase; LDL: Low-Density Lipoprotein; HDL-C: High-Density Lipoprotein; WBC: White Blood Cell; Hgb: Hemoglobin; PLT: Platelet; IL-35: Interleukin-35.

Comparison of serum IL-35 between healthy controls and IMN patients.

We compared the serum IL-35 levels of 22 healthy controls and 174 IMN patients. The results showed that the level of serum IL-35 in IMN patients (whether active disease or in remission) was higher than that in healthy controls (88.90 (75.45, 114.94) pg/ml *Vs*.338.63 (218.95-428.85) pg/ml, $P<0.001$). Clinical features of healthy

controls are shown in **Table S2** and **Figure S1**. Health controls were from our team's students and staff, with an average age of around 26 years old, and 54.5% were female. This table shows that the age and serum IL-35 levels in healthy controls were significantly lower than in patients with IMN. Age, IL-35 these indicators are continuous variables. The gender is a binary variable.

Table S2. Characteristics of healthy controls and IMN patients

	HC (n=22)	IMN (n=174)	*P* Value
Age (years)	26±2	52±13	<0.001
Gender (female)	12 (54.5%)	68 (39.1%)	0.164
IL-35 (pg/ml)	88.90 (75.45, 114.94)	338.63 (218.95-428.85)	<0.001

Abbreviation IL-35: Interleukin-35.

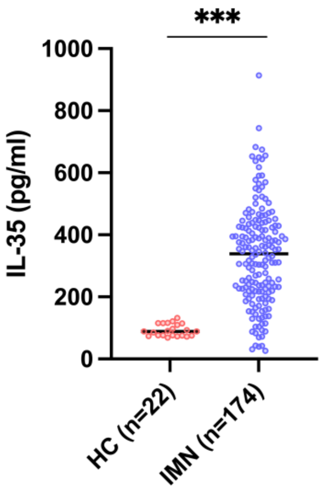

Figure S1 ***represent P<0.001.

Comparison of serum IL-35 between healthy controls (HC, n=22) and IMN patients (n=174). The level of serum IL-35 in healthy controls was significantly lower than that in IMN patients (*P*<0.001). ***represent *P*<0.001.

Correlation analysis between serum IL-35 level and other clinical parameters of IMN

The correlation between IL-35 and various parameters is analyzed by Pearson correlation analysis or Spearman rank correlation analysis according to whether the parameters of correlation analysis conform to normal distribution. See **Table S3** for the analysis results.

It was found that serum levels of IL-35 were positively correlated with ALB (r=0.37, *P*<0.01), remission (r=0.28, *P*=0.01), and negatively correlated with 24hUTP (r=-0.26, *P*=0.02), nephrotic syndrome state (r=-0.39, *P*<0.01), and remission time (r=-0.46, *P*<0.01).

Table S3. Correlation analysis between serum levels of IL-35 and other clinical parameters of IMN

	**Age**	**Gender**	**24hUTP**	**ALB**	**TG**
**Related coefficients (r)**	-0.04	0.17	-0.26	0.37	0.09
***P* Value**	0.70	0.15	0.02	<0.01	0.42

	**CHO**	**Nephrotic syndrome**	**SCr**	**eGFR**	**aPLA2R titer**
**Related coefficients (r)**	-0.23	-0.39	-0.10	0.12	-0.17
***P* Value**	0.05	<0.01	0.37	0.29	0.25

	**Hypertension**	**Diabetes**	**Immunosuppressant**	**Remission**	**Remission time**
**Related coefficients (r)**	0.08	-0.18	0.16	0.28	-0.46
***P* Value**	0.47	0.12	0.18	0.01	<0.01

Negative control of Treg and iTR35 cells in flow cytometry analysis

The expression of Treg and iTR35 in PBMC of IMN patients was detected by flow cytometry (**Figure S2-S3**). Staining procedure is the same as *Flow cytometry analysis in "Materials and Methods"*. The negative control of Treg cells includes blank control and isotype control. The negative control of iTR35 cells includes blank control, isotype control and unstimulated control.

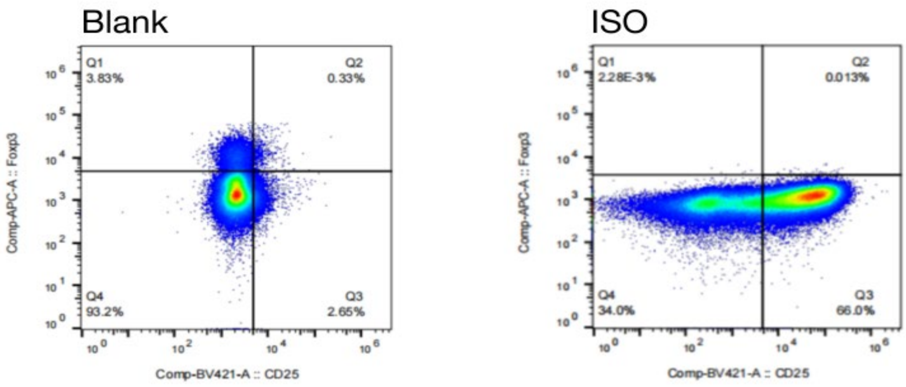

Figure S2

Negative control of Treg cells in flow cytometry analysis, which including blank control and isotype control.

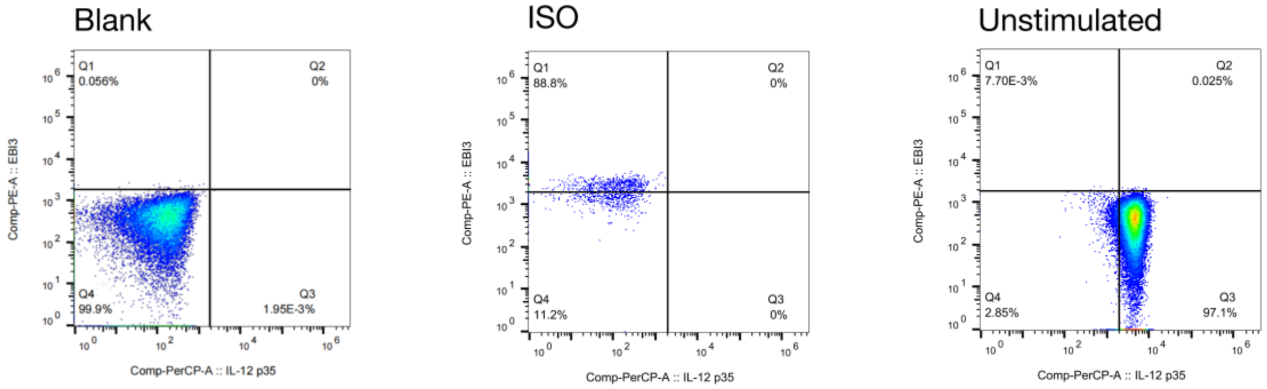

Figure S3

Negative control of iTR35 cells in flow cytometry analysis, which including blank control, isotype control and unstimulated control.
